# The architecture of substrate-engaged TOM–TIM23 supercomplex reveals preprotein proximity sites for mitochondrial protein translocation

**DOI:** 10.1038/s41421-023-00643-y

**Published:** 2024-02-16

**Authors:** Qiang Wang, Jinjin Zhuang, Rui Huang, Zeyuan Guan, Ling Yan, Sixing Hong, Liying Zhang, Can Huang, Zhu Liu, Ping Yin

**Affiliations:** National Key Laboratory of Crop Genetic Improvement, Hubei Hongshan Laboratory, Huazhong Agricultural University, Wuhan, Hubei China

**Keywords:** Cryoelectron microscopy, Protein translocation

Dear Editor,

Mitochondria, housing at least 1000 proteins, play a pivotal role as an organelle, facilitating energy production and metabolite synthesis^1,2^. Mitochondrial proteins are primarily synthesized within the cytosolic ribosome and subsequently transferred to specific mitochondrial compartments through the corresponding translocase complexes, including the translocase of the outer mitochondrial membrane (TOM) and the translocase of the inner mitochondrial membrane (TIM23)^3^. As the main entry gate of mitochondria, the TOM complex is responsible for transferring ~90% of mitochondrial proteins from the cytosol into the mitochondria^1,4^. These mitochondrial proteins are known as preproteins before entering mitochondria. The mitochondrial matrix proteins are the most abundant protein type within mitochondria, and these proteins with N-terminal presequence are transferred into mitochondria successively by both the TOM and TIM23 complexes^1,3^. The TOM and TIM23 complexes are assembled into a transient supercomplex, thus promoting the translocation of preproteins^5^. Although the assembly of the TOM and TIM23 complexes has been elucidated^6–9^, respectively, little is known about how presequence-carrying preproteins pass through the TOM complex.

An artificial substrate Jac1^sfGFP^ (presequence-containing Jac1 fused to superfolder GFP) has been reported to stabilize the TOM–TIM23 supercomplex^5^. To investigate the transfer process of preproteins from cytosol to the mitochondrial matrix, we captured the TOM–TIM23 supercomplex by translocating Jac1^sfGFP^ to isolated yeast mitochondria to obtain TOM–TIM23–Jac1^sfGFP^ supercomplex (Fig. 1a, b). The resultant TOM–TIM23–Jac1^sfGFP^ supercomplex was subsequently subjected to gel filtration chromatography analysis, yielding a protein peak, and this peak presented an ~880-kDa band on blue native PAGE (Supplementary Fig. S1a, b). Mass spectrometry analysis further confirmed that this ~880-kDa band encompassed the expected components, including TOM subunits, TIM23 subunits and Jac1^sfGFP^ (Supplementary Fig. S1c and Table S1). Consequently, we successfully obtained protein samples representing the preprotein-engaged TOM–TIM23 supercomplex.

**Fig. 1:**
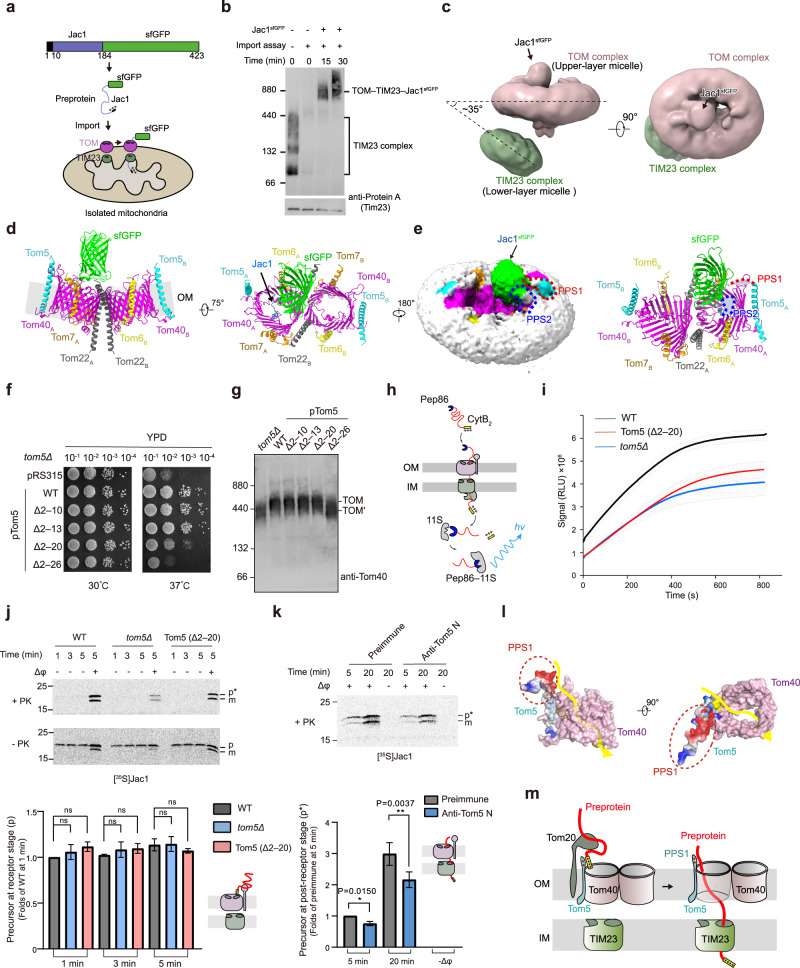
The architecture of substrate-engaged TOM–TIM23 supercomplex. **a** Schematic diagram of the TOM–TIM23–Jac1^sfGFP^ supercomplex capture. **b** Blue native PAGE analysis of the TOM–TIM23–Jac1^sfGFP^ supercomplex. **c** Architecture of the TOM–TIM23–Jac1^sfGFP^ supercomplex. **d**, **e** Structure of the Jac1^sfGFP^-engaged TOM complex. **f** Spot assays of *tom5Δ* strain rescued by Tom5 truncations. **g** Steady-state level of the TOM complex corresponding to **f**. TOM′ representing the TOM complex lacking of Tom5. **h**, **i** Real-time import of the CytB_2_ (220Δ19)–pep86 by NanoLuc assay. **j** Import assays of radiolabeled Jac1 into mitochondria under the indicated conditions. **k** Import assays of radiolabeled Jac1 into mitochondria which were preincubated with rabbit serum against the N-terminus of Tom5. In **j**, **k** data are means ± SEM representing at least three biologically independent samples. *P*-values were calculated by two-tailed Student’s *t*-test. Δφ, membrane potential; p, precursor at the receptor stage; p*, precursor at the post-receptor stage; m, mature Jac1. **l** Potential translocation path for presequence-carrying preproteins. The electrostatic potential surface of full-length Tom5 (predicted by AlphaFold2) was displayed. Red, negatively charged region. **m** Working model of translocation path of the presequence-carrying preproteins across the outer and inner membranes of mitochondria.

After the TOM–TIM23–Jac1^sfGFP^ supercomplex was purified from a 100-L culture of *Saccharomyces cerevisiae*, we further obtained a density map (overall resolution of 4.4 Å) of this supercomplex through cryo-electronic microscopy (cryo-EM) analysis (Supplementary Fig. S1d). This cryo-EM map exhibited the presence of non-parallel double-layer detergent micelles with the upper-layer micelle and the lower-layer micelle corresponding to the TOM complex and the TIM23 complex, respectively (Fig. 1c). The two layers of detergent micelles formed an angle of ~35° in the TOM–TIM23–Jac1^sfGFP^ supercomplex (Fig. 1c). Notably, the density of the TIM23 complex was progressively diminished with the contour level value increasing from 0.08 to 0.16, implying the instability of the TIM23 complex (Supplementary Fig. S2a). Numerous efforts were made including substrate screening and modification, large-scale image collection, and chemical crosslinking to enhance stability of the TIM23 complex to generate a high-resolution map, but no obvious improvements were achieved for the TIM23 map. However, our map presented the densities connecting the upper- and lower-layer micelles, which might be the intermembrane space (IMS) domain of Tom22. These findings suggested the important role of Tom22 in producing the TOM–TIM23 supercomplex (Supplementary Fig. S2b)^10^.

In the architecture of the TOM–TIM23–Jac1^sfGFP^ supercomplex, the structural features of the TOM complex were significantly clearer than those of the TIM23 complex (Supplementary Fig. S2a). Further analysis revealed that the local resolution of the TOM complex structure ranged from 3.0 Å to 6.0 Å (Supplementary Figs. S1d, S3a). The TOM complex consisted of two Tom40 pores, which were surrounded by two copies of three small Tom subunits and tethered by two Tom22 molecules (Supplementary Fig. S3a, b)^6–8^. The densities corresponding to Jac1 polypeptides and sfGFP were observed within the Tom40 channel and on the cytosolic side of the Tom40, respectively (Fig. 1d; Supplementary Fig. S3c–e). The rough densities of the Jac1 polypeptides were mainly distributed close to the cytosolic side of β-strands 10–12 within Tom40 channel, and the remaining densities along β-strands 3–10 (Supplementary Fig. S3f). Moreover, structural analysis of the Jac1^sfGFP^-engaged TOM complex revealed two preproteins proximity sites (PPS) on the mitochondrial surface (Fig. 1d, e; Supplementary Fig. S4a). The PPS1 corresponded to the N-terminus of Tom5 (residues 1–26), while the PPS2 encompassed the loop between β-strands 14 and 15 of Tom40 (Fig. 1e; Supplementary Fig. S4a). The PPS1 and PPS2 were situated on the cytosolic side of the TOM complex, potentially functioning in recognizing the incoming preproteins.

The PPS1 was close to the β-strands 9–11 of the Tom40 (Fig. 1e; Supplementary Fig. S4a). To examine the function of the PPS1, we investigated the growth status of four PPS1-truncated yeast strains (Δ2–10, Δ2–13, Δ2–20, and Δ2–26). We found that the deletion of residues 2–20 or 2–26 of PPS1 resulted in a growth defect at 37 °C (Fig. 1f). The deletion of residues 2–26 affected the steady-state level of TOM complex whose band was slightly lower than the band of the wild-type (WT) mitochondria, but similar to that of *tom5Δ* mitochondria. In contrast, the deletion of residues 2–20 did not affect the steady-state level of the TOM complex (Fig. 1g; Supplementary Fig. S4b). To examine the role of the PPS1 in the preprotein import pathway, we performed import assays using isolated mitochondria. The import efficiency of ^35^S-labeled Jac1 preprotein was slightly lower in Tom5 (Δ2–20) mitochondria than in WT mitochondria (Supplementary Fig. S4a, c). Furthermore, a real-time NanoLuc luciferase assay also demonstrated a decreased import efficiency of modified cytochrome b2 preprotein in Tom5 (Δ2–20) mitochondria (Fig. 1h, i). Superposition analysis of the free TOM complex and Jac1^sfGFP^-engaged TOM complex showed that Tom5 exhibited an ~3-Å movement towards the Tom40 channel in the Jac1^sfGFP^-engaged TOM complex (Supplementary Fig. S4d, e), suggesting that the Tom5 might respond to the incoming substrates. Taken together, these results indicate that the PPS1 might be involved in the import of preproteins to mitochondrial matrix.

Tom5 was identified as a mediator between receptors and the import pore in the preprotein translocation process^11^. Both our crosslinking mass spectrometry assay data and previous report^5^ revealed a crosslink site between the N-terminus of Tom5 (M1) and Jac1 (K172), suggesting the proximity of PPS1 to the preproteins during translocation (Supplementary Fig. S5). Additionally, a photo-crosslinking strategy identified multiple preprotein binding sites within Tom40 (Supplementary Fig. S6)^4,12–14^. Certain binding sites, including the residues 228–234 recognizing pALDH^13^, were close to the PPS1 in Tom5, suggesting that the preproteins might have been delivered from PPS1 to the Tom40 pore. During the translocation process of matrix proteins, precursors first accumulate on the outer membrane, which is known as the receptor stage; then these precursors span both the outer and inner membranes, which is known as the post-receptor stage; finally, they were completely imported into the matrix and become mature proteins (Supplementary Fig. S7a)^11^. The accumulation of Jac1 in Tom5 (Δ2–20) mitochondria exhibited no difference compared to that in WT mitochondria at the receptor stage (Fig. 1j). Further, we disrupted the initial binding of preproteins with Tom5 using antibodies against N-terminus of Tom5^11,15^, and found that both Jac1 precursors at post-receptor stage and mature Jac1 were significantly reduced (Fig. 1k; Supplementary Fig. S7b). Similar results were observed in the Tom5 (Δ2–20) mitochondria (Supplementary Fig. S7c), indicating that the N-terminus of Tom5 is required for preprotein import at the post-receptor stage^11^. The above results jointly indicate that the PPS1 is responsible for guiding and facilitating the delivery of preproteins to the Tom40 channel.

The PPS2, which is not well reconstructed in reported TOM complex structures, has been found to have a domain on the cytosolic side of the TOM complex^6–9^ (Supplementary Fig. S8). PPS2-deleted strains showed no growth defects and did not affect the steady-state level of the TOM complex (Supplementary Fig. S9a–c). Furthermore, the import efficiency of ^35^S-labeled Jac1 exhibited no significant difference between the PPS2-deleted and WT mitochondria, demonstrating that the PPS2 might not be involved in preprotein import (Supplementary Fig. S9d).

The presequence-carrying preproteins have been reported to enter the mitochondria via an “acid chain” pathway involving Tom20, Tom22, Tom5, Tom7, and the negatively charged Tom40 channel^11^. The PPS1 had six conserved negatively charged residues, which might contribute to acid chain formation (Fig. 1l; Supplementary Fig. S5a). Based on these findings, we proposed the following translocation pathway of the presequence-carrying preproteins through the outer and inner mitochondrial membrane: the preproteins are firstly recognized by mitochondrial surface receptors and subsequently delivered to the Tom40 channel under the guidance of PPS1; after passing through the Tom40 channel, they are transported to the mitochondrial matrix by the TIM23 complex (Fig. 1m)^9,14^.

In summary, we reconstructed a preprotein-engaged TOM–TIM23 supercomplex and identified two preprotein proximity sites on the cytosolic side of the TOM complex. Of them, PPS1 was found to be crucial for preprotein import. In a recent study, Zhou et al. found that the preproteins passed the TOM complex through a glutamine-rich region in Tom40 subunit, whereas our study found the preproteins passed through the TOM complex under the guidance of PPS1 on the mitochondrial surface, at an earlier import stage than the tested period in the Zhou’s study^14^. Our findings lay a foundation for further deciphering the mechanism underlying presequence-carrying preprotein transfer across the outer and inner mitochondrial membranes mediated by the TOM and TIM23 complexes.

### Supplementary information

Supplementary information

## Data Availability

The atomic coordinates and EM density map for the reported structure of the TOM–TIM23–Jac1^sfGFP^ supercomplex (PDB: 8W5J; EMDB: EMD-37294) have been deposited in the Protein Data Bank (www.rcsb.org) and the Electron Microscopy Data Bank (www.ebi.ac.uk/pdbe/emdb/), respectively.
