# Supplementary material for: The architecture of substrate-engaged TOM–TIM23 supercomplex reveals preprotein proximity sites for mitochondrial protein translocation
Source: Cell Discov. 2024 Feb 16;10:19. doi: 10.1038/s41421-023-00643-y (PMC10869343; doi:10.1038/s41421-023-00643-y)
Supplement: Supplementary file 1 — Supplementary information [file 41421_2023_643_MOESM1_ESM.pdf]

Supplementary Information for

The architecture of substrate-engaged TOM–TIM23 supercomplex reveals preprotein proximity sites for mitochondrial protein translocation

Qiang Wang[1]†, Jinjin Zhuang[1]†, Rui Huang[1]†, Zeyuan Guan[1], Ling Yan[1], Sixing Hong[1], Liying Zhang[1], Can Huang[1], Zhu Liu[1], and Ping Yin[1]*

Affiliations:
[1]National Key Laboratory of Crop Genetic Improvement, Hubei Hongshan Laboratory, Huazhong Agricultural University, Wuhan 430070, China.

*Correspondence to: Ping Yin (yinping@mail.hzau.edu.cn)
†These three authors contributed equally to this work.

This file includes:

Materials and Methods

Supplementary Fig. S1 to S9

Supplementary Table S1 to S2

Supporting References 1–18

MATERIALS AND METHODS

Yeast strains, plasmids, and antibodies

Variants of yeast strains and plasmids used in this study are listed in Table S1 and Table S2, respectively. The antibodies in this study are summarized in Table S3.

To prepare the yeast TOM–TIM23 supercomplex, the carboxyl terminus of the *TIM23* was fused to tandem affinity purification (TAP) by using a PCR-based homologous recombination strategy[1]. The plasmid pF6Aa-CTAP-KanMX6 was used as the PCR template for TAP. The PCR products were transformed into *Saccharomyces cerevisiae* (strain BY4741a) using the lithium acetate method, allowing homologous recombination. Transformants were selected from the YPD solid medium with Geneticin (Solarbio, G418). The correct insertion of the tag into the genome was confirmed by western blotting.

Deletion of *TOM5* was performed using homologous recombination and linear PCR-amplified DNA fragments as the substrates for transformation. Briefly, for deletion of the *TOM5* gene, a PCR product containing the *HIS3* marker (amplified from pFA6a-HIS3MX6 plasmid) and short sequences homologous to the flanking regions of the *TOM5* locus was used to delete the *TOM5* gene. To isolate positive clones, yeast transformants were grown on the selective medium lacking histidine. The *TOM5*-deletion strain was further verified by sequencing. To generate yeast strains expressing wild-type or mutant Tom5 proteins, the ORF of wild-type and mutant *TOM5* along with their native promoters were cloned onto pRS315 plasmids. These plasmids (including the control plasmid) were subsequently transformed into the *tom5Δ* strain (YS80).

The *TOM40* gene was amplified from genomic DNA using Tom40-316 primers and cloned into pRS316 and pRS315 vectors, respectively. The pRS316-Tom40 plasmid was transformed into the W303-1A yeast strain cells and the endogenous *TOM40*

gene was replaced by a selectable cassette containing the *HIS3* marker and the 40-bp short sequences homologous to the flanking regions of the Tom40 locus. Site-directed mutagenesis was used to generate the mutant plasmids from pRS315-Tom40. The wild-type or mutant plasmids of pRS315-Tom40 were transformed into the strain (*tom40Δ::HIS3 pRS316-Tom40*). The empty pRS315 plasmid was transformed as the vector control. The strains were counter-selected on 5-FOA plates to eliminate the pRS316 plasmid.

In this study, cells were grown in YPD (1% yeast extract, 2% peptone, 2% glucose), YPG (1% yeast extract, 2% peptone, 3% glycerol), YPDG (1% yeast extract, 2% peptone, 3% glycerol, 0.8% glucose), or synthetic dextrose (SD) medium having 2% glucose and 0.66% yeast nitrogen base (YNB) without amino acids and with appropriate supplements. The plating medium was prepared with 2% agarose.

Mitochondria isolation

Yeast strains were grown in 10 ml of YPD precultures and then inoculated into 1-liter YPDG cultures in a shaking incubator for 24 h at 30°C, 210 rpm. Yeast cells were harvested by centrifugation at 900 g for 20 min and then washed with ultrapure water. After incubating in DTT buffer (100 mM Tris-H_2SO_4, pH 9.4, 10 mM DTT) at 30°C for 30 min, yeast cells were centrifuged at 2,000 g at room temperature for 20 minutes. Next, the yeast cells were resuspended in sorbitol buffer (1.2 M sorbitol, 20 mM K_2HPO_4, 5 mM KH_2PO_4, pH 7.2) supplemented with snail enzymes (Solarbio) (40 mg per 1 g yeast cell) and incubated at 37°C for 60 min to digest the yeast cell wall. Spheroplasts were obtained by centrifugation at 2,000 g for 20 min and resuspended in homogenization buffer (600 mM sorbitol, 10 mM Tris-HCl, pH 7.4, 1 mM EDTA, 2 mg ml^{-1} BSA, and 1 mM PMSF). The cells were disrupted with a Dounce homogenizer (Sigma) (20 cycles on ice). Cell debris was removed by two rounds of centrifugations at 2,000 g at 4°C for 10 min. Mitochondria were isolated from the supernatant by centrifugation at 1,7000 g for 15 min at 4°C. Collected mitochondria fraction were resuspended in SEM buffer (250 mM sucrose, 1 mM EDTA, 10 mM

MOPS, pH 7.2) and quantified to approximately 10 mg ml^{-1} (total protein) followed by shock-freezing in liquid nitrogen and stored in aliquots at -80°C.

Expression and purification of $Jac1^{sfGFP}$

The expression and purification of $Jac1^{sfGFP}$ with a C-terminal 8×His tag was carried out using the pET21b vector and expressed in *E. coli* BL21 (DE3). Expression of $Jac1^{sfGFP}$ was induced by adding 0.2 mM IPTG at 16°C for 16 h. When OD_{600} reached ~1.1, the cells were harvested and resuspended in lysis buffer (25 mM Tris-HCl pH 8.0, 150 mM NaCl). Cell disruption was performed using a homogenizer (JNBIO, China) and the resulting cell debris was removed via centrifugation at 23,000 g, 4°C for 60 min. The supernatant was collected and loaded onto Ni-NTA resin (Qiagen). After loading, the resin was washed with 30-bed volumes of wash buffer (25 mM Tris-HCl pH 8.0, 150 mM NaCl, 15 mM imidazole). The bound protein was subsequently eluted using elution buffer (25 mM Tris-HCl pH 8.0, 250 mM imidazole). The eluant was further purified using ion exchange chromatography (Source 15Q 10/100, GE Healthcare). The fractions corresponding to the peak were pooled for import assay.

The capture of TOM–TIM23–$Jac1^{sfGFP}$ supercomplex

The fresh mitochondria were incubated with purified $Jac1^{sfGFP}$ proprotein in import buffer (3% BSA, 250 mM Sucrose, 80 mM KCl, 5 mM $MgCl_2$, 2 mM KH_2PO_4, 5 mM Methionine, 10 mM MOPS/KOH pH 7.2, plus 2 mM ATP, 2mM NADH, 5 mM creatine phosphate and 0.1 mg ml^{-1} creatine kinase, pH 7.4) at 25°C for 30 min. The mitochondria were then reisolated and assessed using SDS-PAGE and blue native PAGE (BN-PAGE) with antibodies against protein A.

For the large-scale purification of TOM–TIM23–$Jac1^{sfGFP}$ supercomplex, a total of 100-L $TIM23^{TAP}$ yeast cells were cultured. After being reisolated from the import reaction, the mitochondria were solubilized in lysis buffer A (150 mM NaCl, 25 mM HEPES pH 7.4, 10% glycerol, 1 mM PMSF, 4 μg ml^{-1} pepstatin A, 4 μg ml^{-1} aprotinin,

and 4 μg ml^{-1} leupeptin) with 1% digitonin for 2 h at 4°C. After centrifugation at 23,000 g for 60 min, the supernatant was mixed with IgG affinity resin (Genscript) at 4°C for 1 h, followed by washing with 30-bed volumes of lysis buffer A with 0.06% digitonin. Then the protein was eluted with lysis buffer A containing 0.06% digitonin and the TEV protease. Finally, the eluant was concentrated using a 100-kDa cut-off Centricon (Millipore) and further analyzed using a Superose 6 increase 10/300 column (GE Healthcare) equilibrated with SEC buffer (25 mM HEPES pH 7.4, 150 mM NaCl, and 0.06% digitonin). The peak fractions were combined and concentrated for further cryo-EM study.

Cryo-EM sample preparation and data acquisition

For cryo-EM sample preparation, 3.5-μl aliquots of the purified yeast TOM–TIM23–$Jac1^{sfGFP}$ at a concentration of ~3.0 mg ml^{-1} were dropped onto glow-discharged holy carbon grids (Quantifoil Au R1.2/1.3,300 mesh), blotted with a vitrobot Mark IV (ThemoFisher Scientific) using 3.5-s blotting time under conditions of 100% humidity at 8°C, and rapidly plunged into liquid ethane that had been pre-cooled by liquid nitrogen. Subsequently, the cryo-EM grids were transferred to a Titan Krios operating at 300 kV, equipped with a GatanK3 Summit detector and a GIF Quantum energy filter.

Images for the TOM–TIM23–$Jac1^{sfGFP}$ were recorded in the counting mode with a magnification of 81,000×, resulting in a pixel size of 1.07 Å. Each image was dose fractionated to 32 frames with a total electron dose of 50 $e^{-}Å^{-2}$ and a total exposure time of 3.5 s. A summit direct electron detector with a slit width of 20 eV on the energy filter was used with a preset defocus range from -1.2 to -2.2 μm. EPU was used for fully automated data collection. All images were motion-corrected using MotionCor2 with a binning factor of 2 with concurrent dose weighting[2]. The defocus values were estimated using Gctf[3].

Cryo-EM data processing

The schematics of the data processing pipeline are shown in supplementary Figure S1. For the TOM–TIM23–Jac1^{sfGFP} supercomplex, a total of 5,150,483 particles were automatically picked using cryoSPARC blob picker from 14,017 manually selected micrographs[4]. After 2D classification, a total of 4,819,029 good particles were selected and subjected to several cycles of 3D classification in cryoSPARC. Particles belonging to the best class (50,070) were selected; this is followed by non-uniform refinement and local refinement. The TOM–TIM23–Jac1^{sfGFP} supercomplex yielded a particle density with an estimated resolution of 4.4 Å based on gold-standard Fourier shell correlation (FSC)[5].

Model building and refinement

For the structure building of TOM–TIM23–Jac1^{sfGFP}, the reported TOM complex structure (PDB ID: 6UCU) was rigidly docked into the density map and manually adjusted using COOT[6]. The TIM23 and Jac1^{sfGFP} were not built due to their poor densities. A lipid was assigned to the density between two Tom40 molecules according to the reported TOM complex structure (PDB ID: 6JNF). The initial fitted model was further refined by iterative rounds of model adjusting in COOT. This resulting model was refined against the map using PHENIX in real space with secondary structure and geometry restraints, respectively[7]. Model quality was evaluated using the Molprobity scores and the Ramachandran plots.

NanoLuc import assay

The NanoLuc assay refers to the method in previously reported with some modifications[8-10]. The 11S protein is the large fragment of an engineered luciferase known as NanoLuc, which can interact with a short fragment called pep86 to form a holo NanoLuc. For expressing 11S in the mitochondria matrix of yeast, we fused the alpha subunit of ATP synthase (ATP1/YBL099W, 1–35 aa) to the N-terminus of the 11S fragment in pYES2 plasmid and added a C-terminal HA tag for detection. Subsequently, the plasmid was transformed into BY4741a strain cells. Mitochondria

isolation and purification methods were performed as previously described.

For the import assay, we used a truncated form of $CytB_2$ consisting of the first 220 residues (with deletions of E43–T65), followed by a myc tag and the pep86 peptide. $CytB_2$ (220Δ19)–pep86 was sub-cloned into the expression vector pET21b and transformed into *E. coli* BL21 (DE3) cells. When OD_{600} reached 0.8, the protein was expressed for 6 h at 37°C upon induction with 1 mM IPTG. Cells were harvested, resuspended, and disrupted in the lysis buffer (25 mM Tris-HCl, 20 mM KCl, 1 mM PMSF, and pH 7.4). After centrifuging at 23,000 g, the inclusion bodies were collected and solubilized in 6 M urea-contained lysis buffer and loaded onto Ni-NTA resin. The bound proteins were also washed with lysis buffer containing 6 M urea. Finally, the bound proteins were eluted with lysis buffer containing 6 M urea and 300 mM imidazole. GST–Dark, an inactive variant of the pep86 peptide, differs from the pep86 sequence by a point mutation at a critical activity arginine residue.

For the NanoLuc import assay, we followed the experimental procedures described in the previous study with some modifications. Briefly, this assay was carried out at 25°C in general white 96-well plates, in an import buffer (containing 3% BSA, 250 mM Sucrose, 80 mM KCl, 5 mM $MgCl_2$, 2 mM KH_2PO_4, 5 mM Methionine,10 mM MOPS/KOH pH 7.2, 2 mM ATP, 2mM NADH, 5 mM creatine phosphate and 0.1 mg ml^{-1} creatine kinase, 20 μM GST–Dark protein, 2 μM Furimazine). 50 μg ml^{-1} of mitochondria were added into the buffer and incubated for 10 min. Then, 10-μl precursors $CytB_2$ (220Δ19)–pep86 were mixed into the plate to initiate the reaction and make the final volume of 120 μl. Finally, the luminescence was measured and collected by EnSpire® Multimode Plate Reader for 1 s per well until the reading value reached a steady state. The experiment was repeated at least three times, and the raw data were subjected to analysis of variance.

Radiolabeled preprotein import assay[11]

For *in vitro* import assay, pGEM-4Z vectors encoding Jac1 were utilized for in vitro

coupled transcription/translation using the SP6 TnT kit (Promega, Cat number: L2080). Radiolabeled preprotein was synthesized *in vitro* using rabbit reticulocyte lysate in the presence of [^{35}S]-Methionine (Perkin-Elmer, Cat number: NEG709A005MC). These radiolabeled preproteins were imported into 50 μg mitochondria per reaction in the presence of 100 μl import buffer (3%BSA, 250 mM Sucrose, 80 mM KCl, 5 mM $MgCl_2$, 2 mM KH_2PO_4, 5 mM Methionine, 10 mM MOPS/KOH pH 7.2, plus 2 mM ATP, 2 mM NADH, 5 mM creatine phosphate and 0.1 mg ml^{-1} creatine kinase, pH 7.4) at 25°C for 2 min, 6 min, and 20 min. Mitochondrial membrane potential was dissipated by adding 2 mM oligomycin and 0.1 mM valinomycin. Non-imported preprotein was digested with 50 μg ml^{-1} proteinase K on ice for 10 min, followed by adding 1 mM PMSF for 10 min on ice to stop the digestion reaction. Then, mitochondria were washed with 500 μl ice-cold SEM buffer (20 mM MOPS/KOH, 250 mM sucrose, 1 mM EDTA, pH 7.2). Finally, mitochondria were solubilized in 30 μl SDS loading buffer and analyzed via SDS-PAGE and the radioactive signals were detected using Typhoon PhosphorImage (GE Healthcare).

For the second *in vitro* import assay, we utilized mitochondria treated with the Tom5 antibody. We prepared IgG directed against the Tom5 N-terminal sequence (MFGLPQQEVSEEEKRAH). Before the import reaction, IgG from preimmune serum or anti-Tom5-N serum was pre-incubated with wild-type mitochondria for 1 h, at a concentration of 200 μg per reaction. Preproteins were synthesized in rabbit reticulocyte lysates in the presence of [^{35}S] methionine, as described above. Import reactions were initiated by adding 5 μl of translated products at 25°C for 5 min and 20 min. As a control, a group of import reactions for 20 min with mitochondrial membrane potential was dispatched by adding 2 mM oligomycin and 0.1 mM valinomycin. The imported preproteins were sedimented by centrifugation and analyzed by SDS-PAGE electrophoresis after import, followed by autoradiography analysis. For quantitative statistical analysis, the quantities of mitochondria and preproteins were standardized across parallel reactions, and the experiment was

repeated four times.

Spot assay

The growth phenotypes of N-terminus-truncations Tom5 yeast cells were analyzed on fermentable (glucose-containing) medium at 30°C and 37°C. The yeast cells were cultivated overnight at 30°C in the SC-His-Leu medium, then diluted to an OD_{600} of 1.0 with fresh SC-His-Leu medium. Subsequently, 10-fold serial dilutions were performed. A 3.5-μl aliquot of each dilution was spotted on YPD plates and then incubated at 30°C and 37°C for approximately 48 h until saturation was achieved.

The growth phenotypes of yeast cells with internal deletions of Tom40 were analyzed in fermentable (glucose-containing) and non-fermentable (glycerol-containing) medium at 30°C and 37°C. The cells were cultivated overnight at 30°C in SC-His-Leu medium, then diluted to an OD_{600} of 1.0 with fresh SC-His-Leu medium, and fourfold serially diluted. Approximately 3.5 μl of each dilution was spotted onto the YPD and YPG plates containing 1 mg ml^{-1} 5-FOA and incubated until saturation at 30°C and 37°C, which took approximately 48 h.

Mass spectrometry analysis

The TOM–TIM23–$Jac1^{sfGFP}$ supercomplex was separated by blue native PAGE[12]. The ~880-kDa band was subjected to in-gel digestion before MS analysis. Specifically, the bands were excised from the gel, treated with 10 mM DTT, and alkylated with 50 mM iodoacetamide. In-gel digestion was then performed with sequencing grade modified trypsin in 50 mM NH_4HCO_3 at 37°C overnight. The peptides were extracted twice with 0.1% trifluoroacetic acid (TFA) in the 50% acetonitrile (ACN) aqueous solution for 30 min. The peptide extracts were then centrifuged in a SpeedVac to reduce the volume. For the LC-MS analysis, peptides were redissolved in 20 μl 0.1% TFA and separated by a 60 min gradient elution at a flow rate of 0.3 μl min^{-1} with a Thermo-Easy nLC1200 system, which was directly interfaced with a Q Exactive HF hybrid quadrupole Orbitrap. The raw data were analyzed by MaxQuant (version

1.6.14) using standard settings against the Saccharomyces cerevisiae FASTA database downloaded from UniProtKB. The LC-MS results are listed in Supplementary Table S1.

The CXMS analysis was conducted with the previous study[13]. Briefly, 10 mM BS^3 was added to the TOM–TIM23–Jac1^{sfGFP} supercomplex (~0.5 µM) at a molar ratio of 20:1. The reaction was performed at room temperature for 30 min and then quenched by the addition of 50 mM NH_4HCO_3. The sample was further treated with 5 mM DTT at 25°C for 10 min and alkylated with 5 mM iodoacetamide in the dark for 30 min. Subsequently, 5 volumes of 50 mM NH_4HCO_3 were added to dilute the sample. Trypsin digestion was carried out at 37°C overnight with sequencing grade modified trypsin (Promega, mass ratio = 1:20). The reaction was quenched with trifluoroacetic acid to a final concentration of 5%. Trypsin-digested peptides were purified with C18 Spin Tips (Thermo Fisher Scientific) and analyzed in the Q Exactive HF Hybrid Quadrupole-Orbitrap mass spectrometer (Thermo Fisher Scientific) coupled to an EASY-nLC 1,200 liquid chromatography system, with a 75 µm, 15 cm Acclaim PepMapTM RSLC column. The peptides were eluted over a 75-minute linear gradient from 95% buffer A (water with 0.1% Formic acid) to 35% buffer B (acetonitrile with 0.1% Formic acid) with a flow rate of 0.3 µl min^{-1}. Each full MS scan (Resolution = 120,000) was followed by 15 data-dependent MS2 (Resolution = 15,000), with a stepped normalized collision energy of 10, 25, and 30. The isolation window was set to 1.6 m z^{-1}. Precursors of charge states 3-6 were collected for MS2 scans. The dynamic exclusion window was set to 40 s. The cross-linking data were analyzed by pLink2. The following search parameters were used: MS1 accuracy of ±20 ppm; MS2 accuracy = ±20 ppm; enzyme = trypsin (with full tryptic specificity but allowing up to three missed cleavages); crosslinker = BS^3 (with an assumed reaction specificity for lysine and protein N termini); fixed modifications = carbamidomethylation on cysteine; variable modifications = oxidation on methionine, hydrolyzed/analyzed BS^3 from reaction with ammonia or water on a free cross-linker end.

Supplementary Table 1. List of yeast strains generated in this study.

Name	Strain	Genotype/Description
	BY4741a	*MATa his3-1 leu2-0 met15-0 ura3-0::*
	W303-1A	*MATa leu2-3,112 trp1-1 can1-100 ura3-1 ade2-1 his3-11,15*
YS22	Tim23^{TAP}	BY4741a *tim23::TAP*
YS80	*tom5*Δ	*MATa leu2-3,112 trp1-1 can1-100 ura3-1 ade2-1 his3-11,15 tom5*$^{\Delta}$*::HIS3*
CH1	Tom40 WT	*MATa leu2-3,112 trp1-1 can1-100 ura3-1 ade2-1 his3-11,15 tom40*$^{\Delta}$*::CgHIS3[pRS315/Tom40]*
CH2	Tom40$^{\Delta 278–287}$	*MATa leu2-3,112 trp1-1 can1-100 ura3-1 ade2-1 his3-11,15 tom40*$^{\Delta}$*::CgHIS3[pRS315/ Tom40*$^{\Delta 278–287}$*]*
CH3	Tom40$^{\Delta 278–295}$	*MATa leu2-3,112 trp1-1 can1-100 ura3-1 ade2-1 his3-11,15 tom40*$^{\Delta}$*::CgHIS3[pRS315/ Tom40*$^{\Delta 278–295}$*]*
CH4	Tom40$^{\Delta 282–292}$	*MATa leu2-3,112 trp1-1 can1-100 ura3-1 ade2-1 his3-11,15 tom40*$^{\Delta}$*::CgHIS3[pRS315/Tom40*$^{\Delta 282–292}$*]*
CH5	Tom40$^{\Delta 283–295}$	*MATa leu2-3,112 trp1-1 can1-100 ura3-1 ade2-1 his3-11,15 tom40*$^{\Delta}$*::CgHIS3[pRS315/ Tom40*$^{\Delta 283–295}$*]*
HB7	tom5$^{\Delta}$ (pRS315)	*MATa leu2-3,112 trp1-1 can1-100 ura3-1 ade2-1 his3-11,15 tom5*$^{\Delta}$*::HIS3[pRS315]*
HB8	Tom5 WT	*MATa leu2-3,112 trp1-1 can1-100 ura3-1 ade2-1 his3-11,15 tom5*$^{\Delta}$*::HIS3[pRS315/Tom5]*
HB9	Tom5$^{\Delta 2–10}$	*MATa leu2-3,112 trp1-1 can1-100 ura3-1 ade2-1 his3-11,15 tom5*$^{\Delta}$*::HIS3[pRS315/Tom5*$^{\Delta 2–10}$*]*
HB10	Tom5$^{\Delta 2–13}$	*MATa leu2-3,112 trp1-1 can1-100 ura3-1 ade2-1 his3-11,15 tom5*$^{\Delta}$*::HIS3[pRS315/Tom5*$^{\Delta 2–13}$*]*
HB11	Tom5$^{\Delta 2–20}$	*MATa leu2-3,112 trp1-1 can1-100 ura3-1 ade2-1 his3-11,15 tom5*$^{\Delta}$*::HIS3[pRS315/Tom5*$^{\Delta 2–20}$*]*
HB12	Tom5$^{\Delta 2–26}$	*MATa leu2-3,112 trp1-1 can1-100 ura3-1 ade2-1 his3-11,15 tom5*$^{\Delta}$*::HIS3[pRS315/Tom5*$^{\Delta 2–26}$*]*

Supplementary Table 2. List of plasmids generated in this study.

Plasmids	Description
HR30	Jac1^{sfGFP} in pET21b (Amp)
WQ33	Tom40(-160, +235) in pRS315 (Amp, LEU2)
WQ34	Tom40(-160, +235) in pRS316 (Amp, URA3)
HR84	Tom40$^{\Delta 282–292}$ in pRS315 (Amp, LEU2)
HR86	Tom40$^{\Delta 283–295}$ in pRS315 (Amp, LEU2)
HR87	Tom40$^{\Delta 278–295}$ in pRS315 (Amp, LEU2)
HR88	Tom40$^{\Delta 278–287}$ in pRS315 (Amp, LEU2)
HR110	Tom5 full length in pRS315 (Amp, LEU2)
HR111	Tom5$^{\Delta 2–10AA}$ in pRS315 (Amp, LEU2)
HR112	Tom5$^{\Delta 2–13AA}$ in pRS315 (Amp, LEU2)
HR113	Tom5$^{\Delta 2–20AA}$ in pRS315 (Amp, LEU2)
HR114	Tom5$^{\Delta 2–26AA}$ in pRS315 (Amp, LEU2)

Supplementary Table 3. List of antibodies used in this study.

**Antibody**	**Source**	**Dilution**
CBP	ABclonal	1:3000
Protein A	Proteintech	1:3000
Tom70	Rabbit polyclonal, self-made	1:3000
Tom40	Rabbit polyclonal, self-made	1:3000
Tom22	Rabbit polyclonal, self-made	1:3000
Sam50	Rabbit polyclonal, self-made	1:1500
Tim44	Mouse monoclonal, self-made	1:3000
Tim23	Rabbit polyclonal, self-made	1:1500
Porin	Rabbit polyclonal, self-made	1:1500

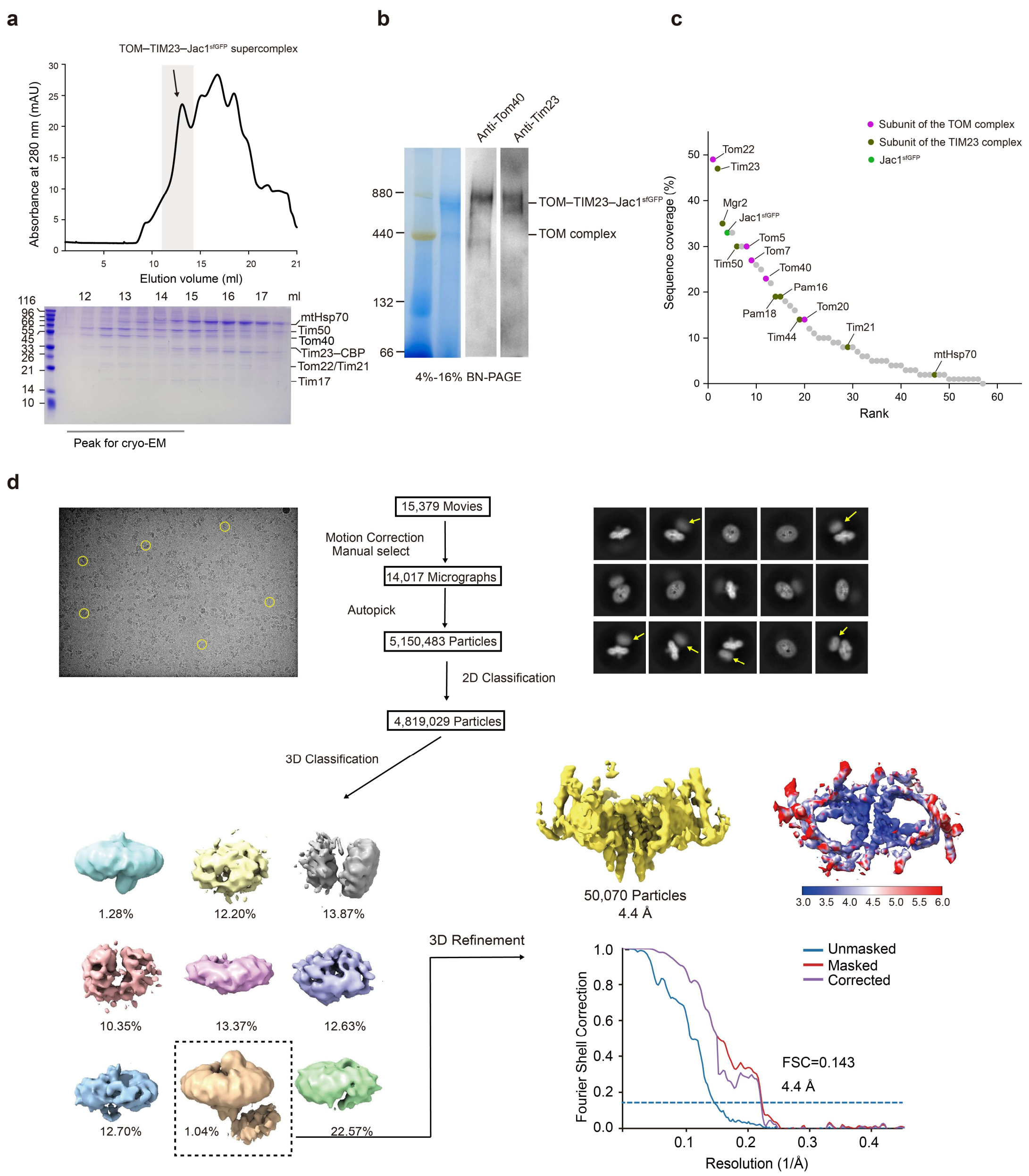

Supplementary Fig. S1. The purification, identification and single particle analysis of the TOM–TIM23–Jac1^{sfGFP} supercomplex. **a** The TOM–TIM23–Jac1^{sfGFP} supercomplex was evaluated using gel filtration chromatography. The peak at around 13.0 ml contained the TOM–TIM23–Jac1^{sfGFP} supercomplex, which was used for cryo-EM analysis shown in d. **b** Blue native PAGE analysis of the TOM–TIM23–Jac1^{sfGFP} supercomplex. **c** Identification of the TOM–TIM23–Jac1^{sfGFP} supercomplex using mass spectrometry. **d** Data collection, image processing, cryo-EM reconstructions, and structural model of the TOM–TIM23–Jac1^{sfGFP} supercomplex. Representative protein particles were highlighted using yellow circles and the lower-layer micelles in the 2D classes were indicated by yellow arrows.

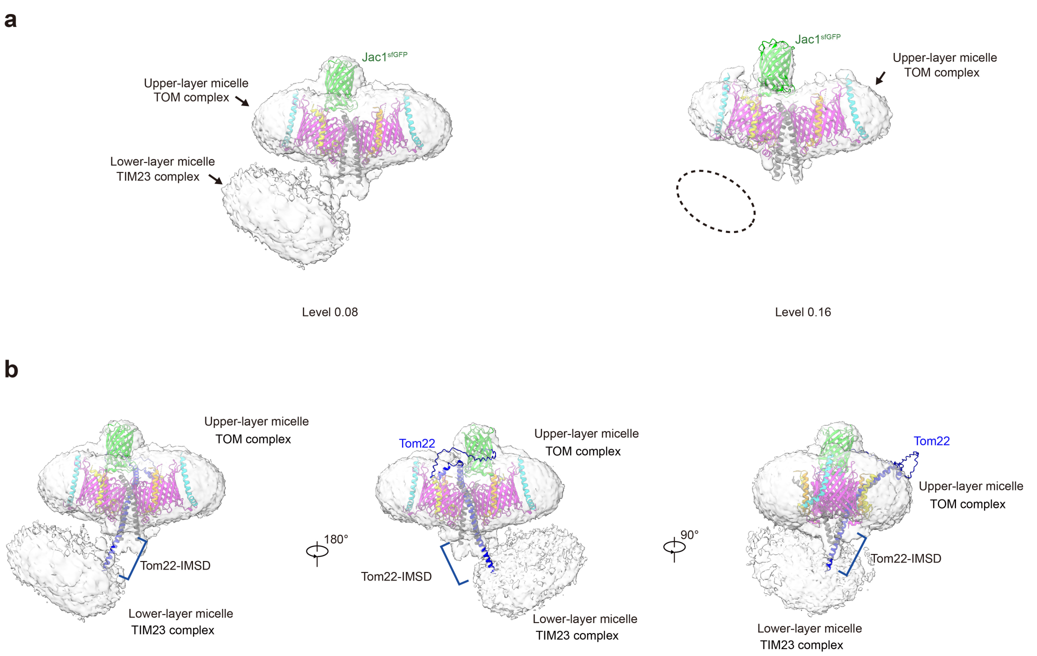

Supplementary Fig. S2. Structural analysis of the TOM–TIM23–Jac1^{sfGFP} supercomplex. a Density of the TIM23 complex was progressively diminished with the contour level value increasing from 0.08 to 0.16. **b** Densities connected to the upper- and lower-layer micelles were observed and highlighted using a blue line. To better represent the density, we docked the AlphaFold2-predicted Tom22 structure into the density map of the TOM–TIM23–Jac1^{sfGFP} complex. Tom22 was depicted in blue color, and the pictures were drawn by ChimeraX.

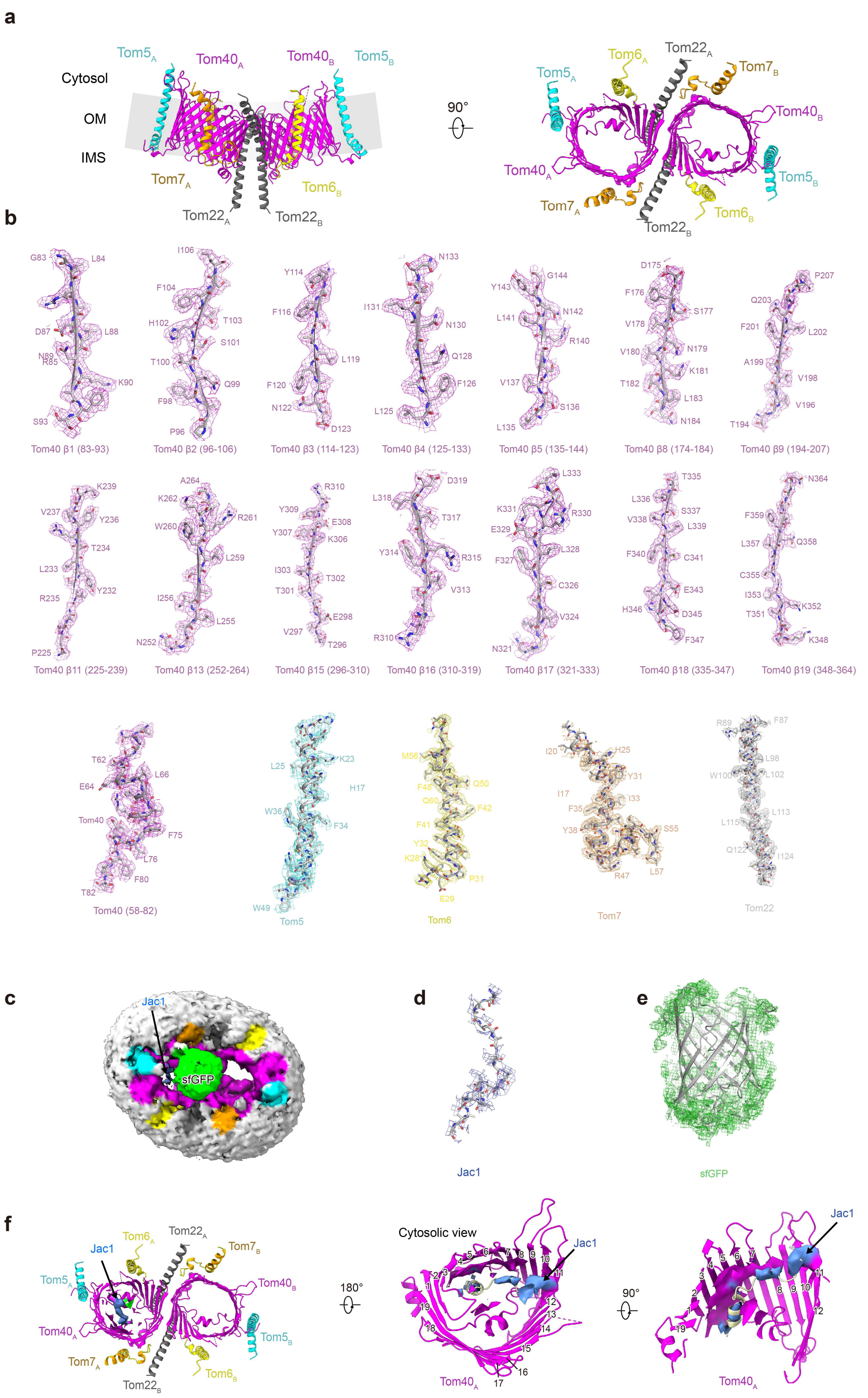

a
Tom5A
Tom40A
Tom40B
Tom5B
Cytosol
OM
IMS
Tom7A
Tom6B
Tom22A
Tom22B
90°
Tom6A
Tom22A
Tom7B
Tom40B
Tom40A
Tom5B
Tom7A
Tom6B
Tom22B
b
Tom40 β1 (83-93)
Tom40 β2 (96-106)
Tom40 β3 (114-123)
Tom40 β4 (125-133)
Tom40 β5 (135-144)
Tom40 β8 (174-184)
Tom40 β9 (194-207)
Tom40 β11 (225-239)
Tom40 β13 (252-264)
Tom40 β15 (296-310)
Tom40 β16 (310-319)
Tom40 β17 (321-333)
Tom40 β18 (335-347)
Tom40 β19 (348-364)
Tom40 (58-82)
Tom5
Tom6
Tom7
Tom22
c
Jac1
sfGFP
d
Jac1
e
sfGFP
f
Tom6A
Tom22A
Jac1
Tom7B
Tom5A
Tom40B
Tom40A
Tom5B
Tom7A
Tom6B
Tom22B
180°
Cytosolic view
Jac1
Tom40A
90°
Jac1
Tom40A

Supplementary Fig. S3. The structures of the Jac1^{sfGFP}-engaged TOM complex. a Structure of the TOM core complex in two perpendicular views. **b** Representative density maps of the indicated segments. All the densities, contoured at 5.5 σ, were prepared in PyMol. **c** Density map of the Jac1^{sfGFP}-engaged TOM complex. **d** and **e** Jac1 and sfGFP with real density map shown in mesh. The densities were contoured at 2.5 σ and 3.0 σ, respectively. **f** Densities of Jac1 were displayed within the TOM core complex model, and difference maps showing the Jac1 densities (slate) superposed on the Tom40 (β-strands were labeled) channel. These pictures were drawn using PyMol and ChimeraX.

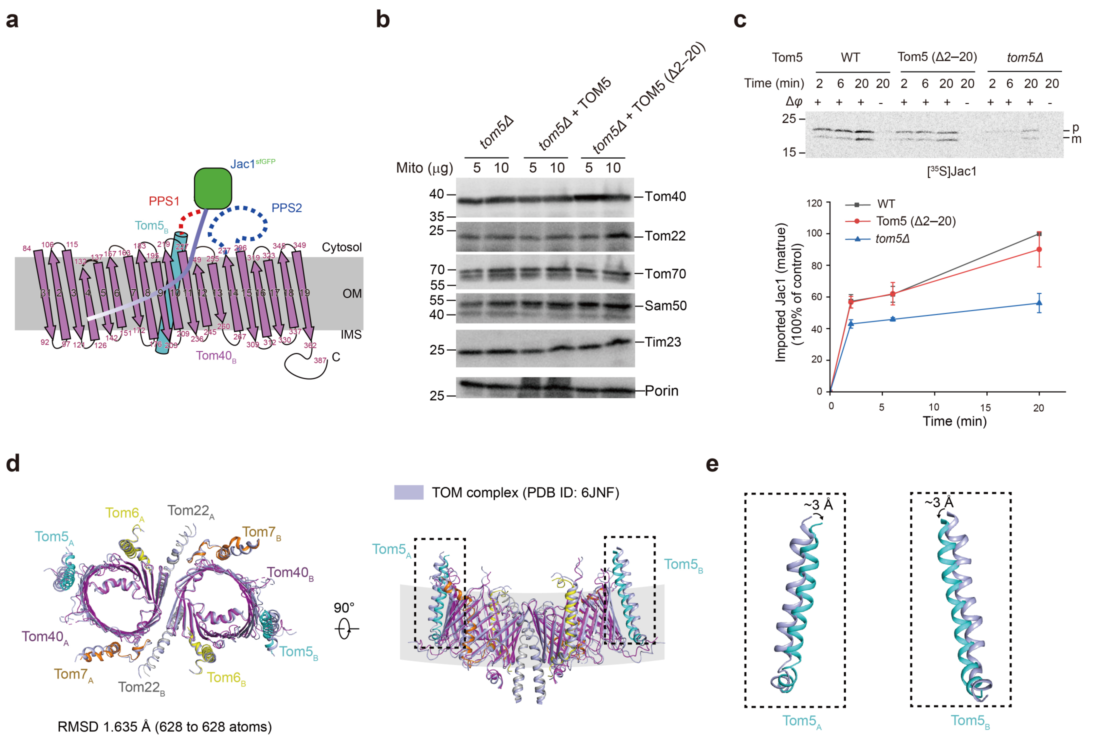

Supplementary Fig. S4. The PPS1 is involved in preprotein translocation. a Schematic diagram of PPS1 and PPS2. Red and blue dashed curves indicate PPS1 and PPS2. The Tom40 and Tom5 were shown in cartoons. **b** Steady level of the indicated mitochondrion. Porin was used as the loading control. **c** Import assays of radiolabeled Jac1 into wild-type, *tom5Δ,* and Tom5 (Δ2–20) mitochondria. Representative data of three independent experiments. Δφ, membrane potential; p, precursor form; m, mature form. **d** Superposition of Jac1^{sfGFP}-engaged TOM complex with TOM complex (PDB ID: 6JNF). **e** The Tom5$_A$ and Tom5$_B$ showed conformational changes, which were indicated by a black dashed rectangular box. These pictures were drawn using PyMol.

a

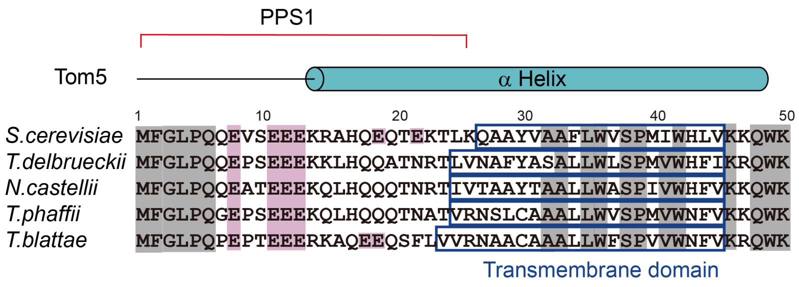

b

CXMS analysis of the TOM–TIM23–Jac1^{sfGFP} complex

Protein 1	Residue	Protein 2	Residue
Tom5	1	Jac1^{sfGFP}	172
Tom5	1	Tom22	2
Tom5	1	Tom6	21
Tim17	55	Tim23	190
Tim17	55	Tim50	108
Tim21	161	Tim50	349
Tim21	164	Tim50	349

Supplementary Fig. S5. The PPS1 has conserved negatively charged residues. a Sequence alignment of Tom5 from yeast strains. This alignment was performed using ClustalW (https://www.genome.jp/tools-bin/clustalw). **b** CXMS analysis of the TOM–TIM23–Jac1^{sfGFP} complex.

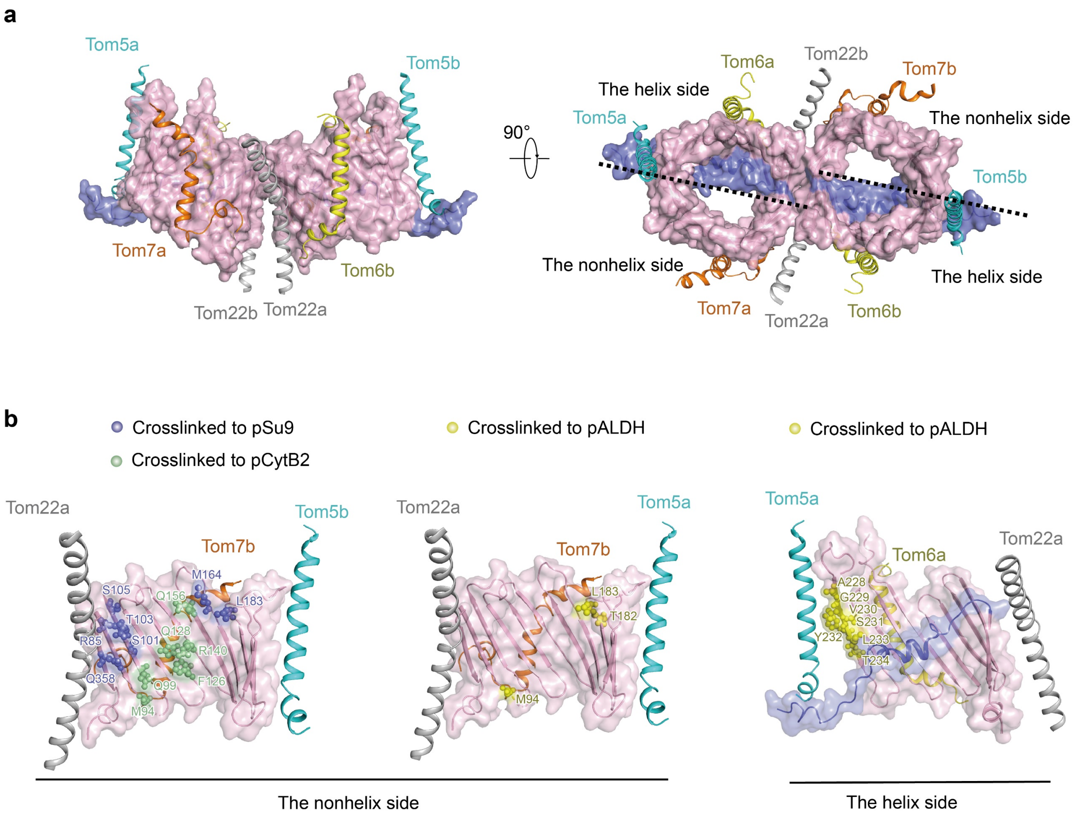

Supplementary Fig. S6. The translocation paths for the presequence-carrying preproteins pass through the interior of the Tom40. a Helix side and nonhelix side of Tom40 were shown on the surface. **b** Residues within Tom40 crosslinked to the translocation intermediates of presequence-carrying preproteins. The crosslinked patches were indicated by colored spheres. These pictures were drawn using PyMol.

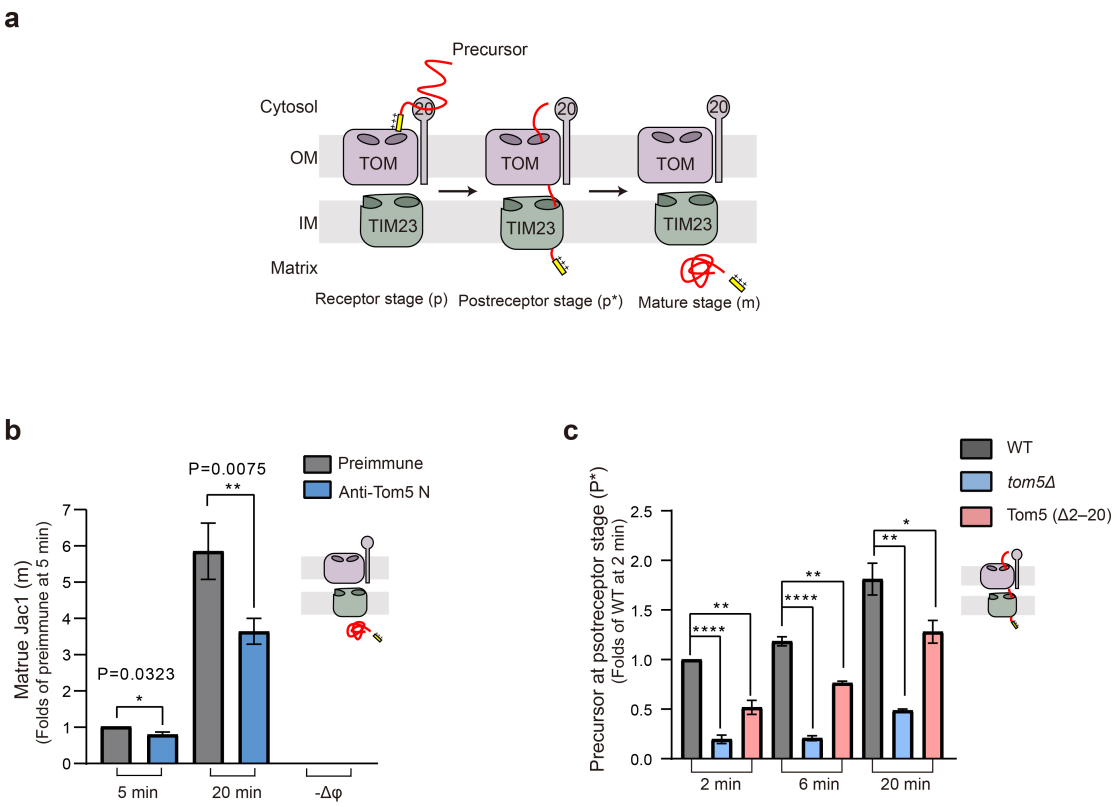

Supplementary Fig. S7. The PPS1 is required for the delivery of preprotein to Tom40. a Schematic diagram of the translocation of presequence-carrying preproteins. **b** Import assays of radiolabeled Jac1 into 50-μg mitochondria which preincubated with rabbit serum against the N-terminus of Tom5. A preimmune serum was used as a control. Statistics of the protein level of Jac1 precursor at the mature stage. **c** Statistics of the protein level of Jac1 precursor at postreceptor stage related to Supplementary Fig. S4c. Data are mean ± SEM representing six biologically independent samples. P values were calculated from the two-tailed Student's t-test. Δφ, membrane potential; p, precursor at receptor stage (precursors can be digested by PK); p*, precursor at postreceptor stage (precursors cannot be digested by PK); m, mature Jac1.

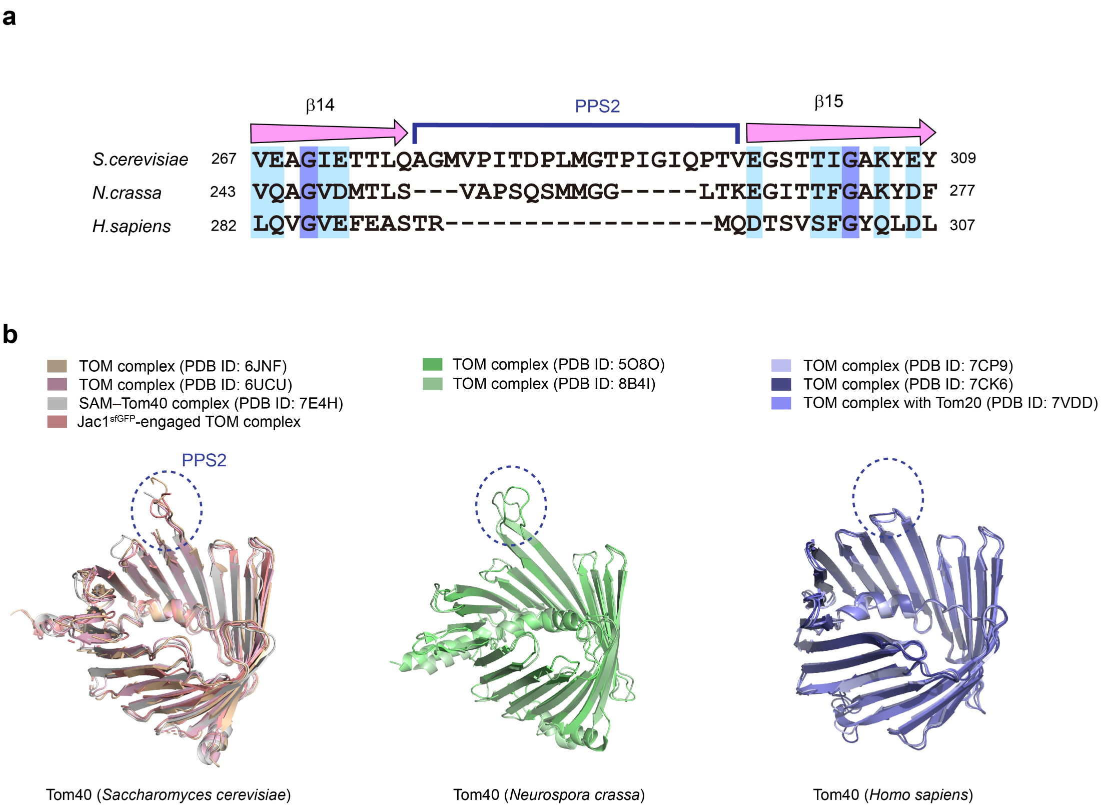

Supplementary Fig. S8. The loop between β14 and β15 of Tom40. a The sequence alignment of the β14 to β15 of Tom40 using ClustalW (https://www.genome.jp/tools-bin/clustalw). The yeast Tom40 contains a ~20-residue loop. The Tom40 in *Neurospora crassa* carries a 10-residue loop. The loop in human Tom40 only contains 4 residues. **b** The loops between β14 to β15 of Tom40 in three kinds of species are indicated by blue dashed circles[14-18]. The PPS2 is marked.

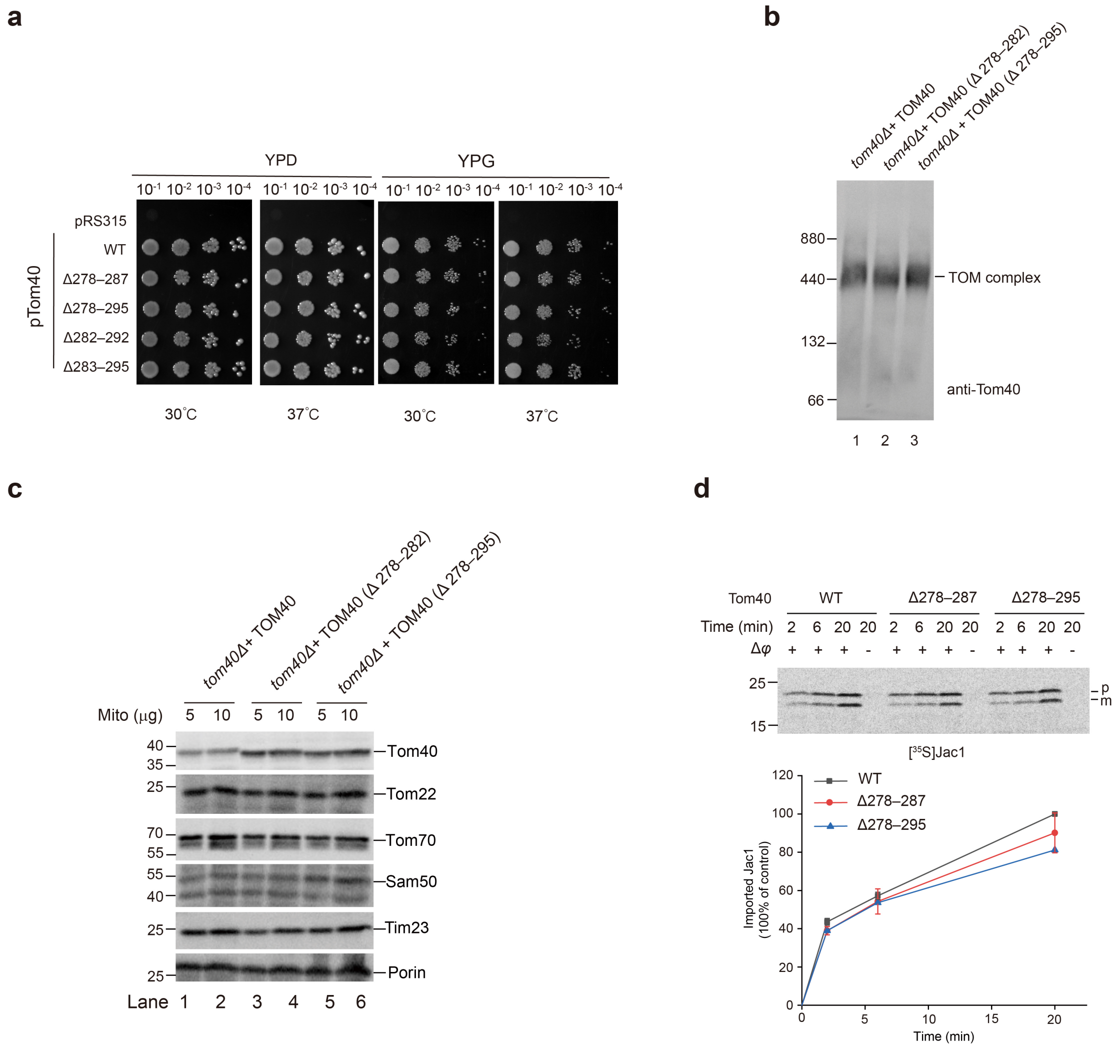

Supplementary Fig. S9. The PPS2 might not be involved in preprotein transporting. **a** Growth status analysis of yeast strains expressing Tom40 variants with the indicated internal deletions on both fermentable and non-fermentable medium. Data are representative of three independent experiments. WT, wild-type. pRS315, empty vector. **b** Steady level of the TOM complex in the mitochondria contained Tom40 variants with the indicated internal deletions. **c** Steady level of the indicated proteins in the mitochondria as in **b**). **d** Import of the ^{35}S-labeled Jac1 in the mitochondria contained Tom40 variants with the indicated internal deletions. Representative data of three independent experiments. Δφ, membrane potential; p, precursor form; m, mature form; WT, wild-type; Δ278–287 and Δ278–29, Tom40 variants.

Supplementary Table S1. Summary of mass spectrometry analysis for the yeast TOM–TIM23–Jac1^{sfGFP} complex

Band (BN-PAGE)	Protein	PSMs	Sequence coverage (%)	Unique peptide
	Tom22	13	49	6
	Tim23	6	47	6
	Mgr2	2	35	2
	Jac1^{sfGFP}	16	33	12
	Tim50	22	30	13
TOM–TIM23–Jac1^{sfGFP} (related to Supplementary Fig. S1c)	Tom5	2	30	1
	Tom7	4	27	2
	Tom40	11	23	7
	Pam18	4	19	4
	Pam16	1	19	1
	Tim44	7	14	5
	Tom20	3	14	2
	Tim21	2	8	2
	mtHsp70	1	2	1

PSM, Peptide spectrum matches.

Supplementary Table S2. Cryo-EM data collection and refinement statistics

**Data Collection and Processing**	TOM–TIM23 supercomplex<br>EMD-37294<br>PDB: 8W5J
Microscope	Krios
Voltage (keV)	300
Camera	Gatan K3
Magnification	81,000
Pixel size at detector (Å/pixel)	1.07
Total electron exposure (e^-/Å^2)	50
Exposure rate (e^-/pixel/sec)	12.48
Number of frames collected during exposure	32
Defocus range (μm)	-1.2~-2.2
Automation software	EPU
Energy filter slit width (eV)	20
Micrographs collected (no.)	15,379
Micrographs used (no.)	14,017
Total extracted particles (no.)	5,150,483
**For each reconstruction:**	
Refined particles (no.)	50,070
Final particles (no.)	50,070
Point-group	C1
Resolution (global, Å)	
- FSC 0.5 (unmasked/masked)	8.0/6.4
- FSC 0.143 (unmasked/masked)	6.7/4.4
Resolution range (local, Å)	3.0-6.0
Map sharpening B factor (Å2)	145.9
Map sharpening methods	Half-maps correlation
**Model composition**	
Protein	942
Ligands/RNA/DNA	1
**Model Refinement**	
Refinement package	PHENIX
- real or reciprocal space	Real space
- resolution cutoff	4.4
Model-Map scores	
- CC	0.79
B factors (Å2)	
Protein residues	185.47
Ligands/RNA/DNA	210.82

R.m.s. deviations from ideal values	
Bond lengths (Å)	0.002
Bond angles (°)	0.629
**Validation**	
MolProbity score	1.66
CaBLAM outliers	1.34
Clashscore	13.63
Poor rotamers (%)	0
C-beta deviations	0
Ramachandran plot	
Favored (%)	97.93
Outliers (%)	0

Supporting references

1. Yan C, Wan R, Bai R, Huang G, Shi Y. Structure of a yeast activated spliceosome at 3.5 Å resolution. *Science* **353**, 904–912 (2016).

2. Zheng SQ, Palovcak E, Armache JP, Verba KA, Cheng Y, Agard DA. MotionCor2: Anisotropic correction of beam-induced motion for improved cryo-electron microscopy. *Nat. Methods* **14**, 331–332 (2017).

3. Zhang K. Gctf: Real-time CTF determination and correction. *J. Struct. Biol.* **193**, 1–12 (2016).

4. Punjani A, Rubinstein JL, Fleet DJ, Brubaker MA. CryoSPARC: Algorithms for rapid unsupervised cryo-EM structure determination. *Nat. Methods* **14**, 290–296 (2017).

5. Rosenthal PB, Henderson R. Optimal determination of particle orientation, absolute hand, and contrast loss in single-particle electron cryomicroscopy. *J. Mol. Biol.* **333**, 721–745 (2003).

6. Emsley P, Cowtan K. Coot: model-building tools for molecular graphics. *Acta. Crystallogr. Sect. D. Biol. Crystallogr.* **60**, 2126–2132 (2004).

7. Adams PD, Afonine PV., Bunkóczi G. PHENIX: A comprehensive Python-based system for macromolecular structure solution. *Acta. Crystallogr. Sect. D. Biol. Crystallogr.* **66**, 213–221 (2010).

8. Pereira GC, Allen WJ, Watkins DW. A High-Resolution Luminescent Assay for Rapid and Continuous Monitoring of Protein Translocation across Biological Membranes. *J. Mol. Biol.* **431**, 1689–1699 (2019).

9. Needs HI, Lorriman JS, Pereira GC, Henley JM, Collinson I. The MitoLuc Assay System for Accurate Real-Time Monitoring of Mitochondrial Protein Import Within Mammalian Cells. *J. Mol. Biol.* **435**, (2023).

10. Hall MP, Unch J, Binkowski B. Engineered luciferase reporter from a deep sea shrimp utilizing a novel imidazopyrazinone substrate. *ACS Chem. Biol.* **7**, 1848–1857 (2012).

11. Priesnitz C, Pfanner N, Becker T. Studying protein import into mitochondria. *Methods Cell Biol.* **155**, 45–79 (2020).

12. Wittig I, Braun HP, Schägger H. Blue native PAGE. *Nat. Protoc.* **1**, 418–428 (2006).

13. Yan J, Liu F, Guan Z, et al. Structural insights into DNA N6-adenine methylation by the MTA1 complex. *Cell Discov.* **9**, (2023).

14. Bausewein T, Mills DJ, Langer JD, Nitschke B, Nussberger S, Kühlbrandt W. Cryo-EM Structure of the TOM Core Complex from Neurospora crassa. *Cell* **170**, 693-700 (2017).

15. Ornelas P, Bausewein T, Martin J, Morgner N, Nussberger S, Kühlbrandt W. Two conformations of the Tom20 preprotein receptor in the TOM holo complex. *Proc. Natl. Acad. Sci.* **120**, e2301447120 (2023).

16. Guan Z, Yan L, Wang Q, et al. Structural insights into assembly of human mitochondrial translocase TOM complex. *Cell Discov.* **7**, 22 (2021).

17. Su J, Liu D, Yang F, et al. Structural basis of Tom20 and Tom22 cytosolic domains as the human TOM complex receptors. *Proc. Natl. Acad. Sci.* **119**, e2200158119 (2022).

18. Wang W, Chen X, Zhang L, et al. Atomic structure of human TOM core complex. *Cell Discov.* **6**, 67 (2020).
